# A novel potential role of pituitary gonadotropins in the pathogenesis of human colorectal cancer

**DOI:** 10.1371/journal.pone.0189337

**Published:** 2018-03-01

**Authors:** Wojciech Marlicz, Agata Poniewierska-Baran, Sylwia Rzeszotek, Rafał Bartoszewski, Karolina Skonieczna-Żydecka, Teresa Starzyńska, Mariusz Z. Ratajczak

**Affiliations:** 1 Department of Gastroenterology, Pomeranian Medical University, Szczecin, Poland; 2 Department of Physiology, Pomeranian Medical University in Szczecin, Szczecin, Poland; 3 Department of Immunology, Faculty of Biology, University of Szczecin, Szczecin, Poland; 4 Department of Histology and Embryology, Pomeranian Medical University, Szczecin, Poland; 5 Department of Biology and Pharmaceutical Botany, Medical University of Gdansk, Gdansk, Poland; 6 Department of Biochemistry and Human Nutrition, Pomeranian Medical University, Szczecin, Poland; 7 Stem Cell Institute, James Graham Brown Cancer Center, University of Louisville, Louisville, United States of America; 8 Department of Regenerative Medicine, Warsaw Medical University, Warsaw, Poland; McGill University, CANADA

## Abstract

**Background:**

Colorectal cancer (CRC) is a leading cause of death in the western world, and its incidence increases with patient age. It is also known that with age there occur changes in the levels of certain hormones, including an increase in the secretion of pituitary gonadotropins (PtGs) as a result of the loss of gonadal hormone feedback. We recently reported that functional PtG receptors are expressed in human lung cancer cells, rhabdomyosarcoma cells, and malignant hematopoietic stem cells.

**Findings:**

Here we report for the first time that the receptors for follicle-stimulating hormone (FSH) and luteinizing hormone (LH) are expressed in primary tumor samples isolated from CRC patients as well as in the established human CRC cell lines HTC116 and HTB37. Moreover, we also report that PtGs stimulate chemotaxis, adhesion, and proliferation of these cell lines.

**Conclusions:**

Our results suggest that PtGs play an important and underappreciated role in CRC pathogenesis, and we call for further studies to better define their role in gastrointestinal malignancies and their direct effect on putative CRC cancer stem cells.

## Introduction

Colorectal cancer (CRC) is one of the most common cancers in western countries. Current concepts concerning its pathogenesis revolve around stem cells (SCs) and innate immunity alterations [1,2], and numerous intrinsic and extrinsic factors have been proposed as contributing to the development of this malignancy [3,4]. The American Cancer Society suggests that the overall lifetime risk of developing CRC is about 1 in 20, with slightly lower risk in women than in men [5]. Currently more than 90% of CRCs occur in people in their sixth and seventh decade of life and older [6].

Importantly, pre-menopausal women have significantly lower risk of developing CRC than age-matched men [7,8], which is in contrast to older, post-menopausal females, who have a worse overall survival prognosis than their male counterparts of similar age [9,10]. As we previously hypothesized, this finding may reflect a higher level of PtGs, such as follicle-stimulating hormone (FSH), observed in postmenopausal women in response to a decrease in secretion of gonadal sex hormones and gonadal dysfunction [11].

Interestingly, it has been reported that the risk of CRC development and progression decreases in postmenopausal women with estrogen or combined estrogen-plus-progestin hormonal therapies [12,13]. This finding is potentially explained by negative feedback of these hormones upon release of pituitary glycoprotiens. To address this issue, we focused our research on the effect of PtGs and studied, in addition to FSH, the effects of luteinizing hormone (LH) and prolactin (PRL) on colorectal cancer (CRC) cell lines. All of these PtGs are potent mitogens, and their role has already been associated with other human malignancies, including prostate [14], breast [15], lung [16], and ovarian cancer [17] as well as certain sarcomas [18].

For example, it has been reported that the use of gonadotropin-based drugs to treat infertility is associated with increased occurrence of ovarian cancer in women, and, by contrast, the use of drugs lowering basal levels of gonadotropins reduces this risk [19]. Similarly, functional expression of FSH and LH receptors in established breast cancer cell lines has shown that sex hormones (SexHs) regulate breast cancer cell motility, adhesion, and invasion [20]. Moreover, functional receptors for pituitary gonadotropins and gonadal SexHs were identified on the surface of human lung cancer cells [16], rhabdomyosarcoma cells [21], and leukemia cells [22].

All of these observations prompted us to elucidate the role of PtGs in CRC, and to address this issue we performed studies with patient samples isolated from primary CRC tumors as well as established human CRC cell lines. Here we report that several SexH receptors are expressed by CRC cells isolated from patient colonic biopsies and the established human CRC cell lines HTC116 and HTB37. Both of these cell lines responded to stimulation by gonadal SexHs by increased adhesion and chemotaxis, resulting from activation of signaling pathways through the corresponding SexH receptors.

Our results may shed more light on the role of PtGs in CRC pathogenesis and open up new diagnostic and therapeutic avenues. The latter possibility will move closer to reality as new drugs with the potential to modulate PtG plasma levels become available [23].

## Materials and methods

### Patient samples

This study was approved by Pomeranian Medical University’s Bioethics Committee and was conducted according to the principles expressed in the Declaration of Helsinki. Frozen primary tumor colon cancer specimens (n = 7) were used to detect the expression of PtGs and gonadal SexH receptors. Tissue samples were obtained from patients during diagnostic colonoscopy after obtaining their written consent. All patients were newly diagnosed with colorectal adenocarcinoma G2. Total RNA was extracted from primary tumors using the RNeasy Mini kit (Qiagen Inc., Valencia CA, USA), including treatment with DNase I (Qiagen). All patients underwent subsequent treatment according to current oncological guidelines and remained under follow-up observation thereafter. The staging of the disease, according to the World Health Organization classification, and survival time were recorded.

### Cell lines

Human CRC cell lines HTC116 (ATCC^®^ CCL-247^™^) and HTB37 (ATCC HTB-37^™^) were cultured in Dulbecco's modified Eagle's medium (DMEM) supplemented with 100 IU/ml penicillin and 10 μg/ml streptomycin with 10% heat-inactivated, non-charcoal-stripped FBS (Sigma-Aldrich, Cat. No. F9665). The cells were cultured in a humidified atmosphere at 37°C in 5% CO_2_.

### Hormones

All hormones were prepared according to the manufacturer’s recommendations. Lyophilisates were dissolved in the substances recommended in the product specification [24–26] and based on previous experience [21, 22, 27] and then added to the culture medium:

1. FSH (R&D Systems, Cat. No. 5925-FS-010) and LH (R&D Systems, Cat. No. 8899-LH-010) were dissolved in sterile PBS containing bovine serum albumin (0.1% BSA) as a carrier protein.
2. Estrogen (Sigma-Aldrich, Cat. No. E2758), progesterone (Sigma-Aldrich, Cat. No. P8783), and danazol (androgen, Sigma-Aldrich, Cat. No. D8399) were dissolved in absolute ethanol (EtOH) and then in sterile culture medium. The final concentration of EtOH in medium with estrogen, progesterone, and danazol was 0.05%, 0.1%, and 0.16%, respectively. The final EtOH concentration in each well was ~0.1%, which is lower than the toxic level and was evaluated in comparison with vehicle controls.
3. Prolactin (Sigma-Aldrich, Cat. No. L4021) was dissolved in 4 mM HCl containing 0.1% BSA as a carrier protein. The final concentration of HCl in the medium with prolactin was 80 μM.

In order to minimize the potential toxicity related to hormone treatments and at the same time maximize hormone stimulation of cell migration, the optimal hormone doses used in experiments were established experimentally according to dose–response assays and toxicity tests. Based on these observations, we used the following doses in experiments: FSH (1 and 10 IU/ml), LH (1 and 10 IU/ml), estrogen (100 nM), progesterone (100 nM), danazol (80 μg/ml), and prolactin (0.5 μg/ml).

### Chemotaxis assay

Chemotaxis assays were performed in a modified Boyden's chamber with 8-μm polycarbonate membrane inserts (Costar Transwell; Corning Incorporated Life Sciences, Lowell, MA, USA) as previously described. In brief, cells detached with 0.25% trypsin were seeded into the upper chambers of inserts at a density of 4 x 10^4^ in 120 μl. The lower chambers were filled with pre-warmed culture medium containing hormones: FSH (10 IU/ml), LH (10 IU/ml), estrogen (100 nM), progesterone (100 nM), danazol (80 μg/ml), or prolactin (0.5 μg/ml). Medium supplemented with 0.5% BSA was used as a negative control. After 24 h, the inserts were removed from the Transwell supports. The cells that had not migrated were scraped off with cotton wool from the upper membrane, and the cells that had transmigrated to the lower side of the membrane were fixed and stained with Hema 3 fixative (Fisher Fisher Scientific) and counted on the lower side of the membrane using an inverted microscope.

### Fibronectin adhesion assay

Ninety-six-well plates were coated with fibronectin (10 μg/ml) overnight at 4°C and blocked with 0.5% BSA for 1 h before the experiment. Cells were made quiescent for 3 h in medium with 0.5% BSA and then incubated for 5 min with hormones: FSH (10 IU/ml), LH (10 IU/ml), estrogen (100 nM), progesterone (100 nM), danazol (80 μg/ml), or prolactin (0.5 μg/ml). Subsequently, cell suspensions (2 x 10^3^/100 μl) were added directly to 96-well plates coated with fibronectin and incubated. After 5 min at 37°C the plates were vigorously washed three times to remove non-adherent cells, and the adherent cells were counted using an inverted microscope.

### Cell proliferation

Cells were grown in 24-well culture plates at an initial density of 1.0 x 10^4^ cells/well. After 24 h the medium was changed to new medium supplemented only with hormones: FSH (1 or 10 IU/ml), LH (1 or 10 IU/ml), estrogen (100 nM), progesterone (100 nM), danazol (80 μg/ml), or prolactin (0.5 μg/ml), and clean medium without any supplements was used as a control. The cell number was gated and calculated at 24, 48, and 72 h by flow cytometry (FACS). In order to avoid a potential decrease in cell viability during 48- and 78-h incubations, we optimized the number of cells plated for proliferation assays as well as the assay conditions. For this purpose the medium was replaced every 24 h. Furthermore, in order to avoid serum-related hormone suppression, both treated cells and their respective controls were assayed in serum-free medium, which resulted in slower cell growth. All data obtained were related to the control sample at the 0-h time point (100%) and measure the proliferation of cancer cells up to 72 h. At the indicated time points, cancer cells were harvested from 24-well culture plates by trypsinization in trypsin-EDTA (0.25%) and scored by FACS analysis using a flow cytometer.

### Conventional RT-PCR

Total RNA from various cells was isolated using the RNeasy Mini kit (Qiagen Inc., Valencia CA, USA), including treatment with DNase I (Qiagen). The RNA was reverse-transcribed with Taqman Reverse Transcription reagents (Applied Biosystems, Grand Island, NY, USA) using the necessary reagents for reverse transcriptase PCR (RT-PCR). Random hexamers, oligo d(T)16, and reverse primers were included for cDNA synthesis. The resulting cDNA fragments were amplified (1 cycle of 8 min at 95°C; 2 cycles of 2 min at 95°C, 1 min at 60°C, 1 min at 72°C; and subsequently 40 cycles of 30 sec at 95°C, 1 min at 60°C, 1 min at 72°C; and 1 cycle of 10 min at 72°C) using Amplitaq Gold polymerase with sequence-specific primers designed using the NCBI Primer-Blast program. One primer in each pair was designed to include an exon-–intron boundary: β-actin (F, *GGATGCAGAAGGAGATCACTG* and R, *CGATCCACACGGAGTACTTG*); hFSH-R (F, *GCTTCTGAGATCTGTGGAGGTT* and R, *ACCTCAGTTCAATGGCATTCCT*); hLH-R (F, *GGGCCGCACTCAGAGG* and R, *AGGGAGGTAGGCAAGTGATAGTC*); hESTRα-R (F, *AGGTGCCCTACTACCTGGAG* and R, *CGGTCTTTTCGTATCCCACCT*); hESTRβ-R (F, *TTTTTGGACACCCACTCCCC* and R, *CACCTGTTGAGGAAAGCGAG*); hANDR-R (F, *CGACTTCACCGCACCTGATG* and R, *CTTCTGTTTCCCTTCAGCGG*); hPROG-R (F, *CGGACACCTTGCCTGAAGTT* and R, *AGTCCGCTGTCCTTTTCTGG*); hPRL-R (F, *GAGCTTCTTCTCACAGAGCCA* and R, *AAGTTCACTTCAGGGTTCATGTGG*).

### Signal transduction studies

Experiments were performed as previously described [22]. Briefly HTC116 cells were stimulated with either 0.5% BSA in Roswell Park Memorial Institute (RPMI) 1640 medium or SexHs for 5 min at 37°C followed by lysis with the RIPA Lysis Buffer System supplemented with protease and phosphatase inhibitors: Na-orthovanadate, PMSF, and PIC (Santa Cruz Biotechnology, Cat. No. sc-24948). The extracted proteins were separated on a 4–12% SDS-PAGE gel and transferred to a PVDF membrane. Phosphorylation of the intracellular kinase p42/44 mitogen-activated protein kinase (p42/44 MAPK) and AKT was detected by phosphospecific p42/44 MAPK (clone 9101) and phosphospecific AKT (Ser473; clone 9271) rabbit polyclonal antibodies (Cell Signaling), respectively. Horseradish peroxidase (HRP)-conjugated goat anti-rabit IgG was used as a secondary antibody (Santa Cruz Biotechnology). To ensure equal protein loading in all lanes, blots were stripped using stripping buffer (Thermo Scientific), and then reprobed with appropriate anti-rabbit p42/44 MAPK (clone 9102) and anti-rabbit AKT (clone 9272) monoclonal antibodies (Cell Signaling). All membranes were treated with enhanced chemiluminescence (ECL) reagent (Amersham Life Sciences), dried, and subsequently exposed to film (Hyperfilm, Amersham Life Sciences). For band visualization, an automatic film processor supplied with fresh warm developer and fixer solutions was used.

### Fluorescence staining

HTC116 cells were fixed in 4% paraformaldehyde for 15 min, permeabilized with 0.1% Triton X100 for 10 min, washed in PBS, pre-blocked with 2.5% BSA in PBS, and subsequently stained for 2 h with antibodies to follicle-stimulating hormone receptor (FSH-R, 1:200, rabbit polyclonal antibody; **Cat. No. sc-13935,** Santa Cruz Biotechnology), luteinizing hormone/choriogonadotropin receptor (LH-R, 1:200, rabbit polyclonal antibody; **Cat. No. sc-25828,** Santa Cruz Biotechnology), androgen receptor (Ab-2, 1:200, rabbit polyclonal antibody; **Cat. No. RB-1358,** Thermo Scientific), and estrogen receptor (ER, Ab-11, 1:200, mouse monoclonal IgG antibody; **Cat. No. MS-354,** Thermo Scientific). Antibodies were diluted in antibody diluents (DAKO). Appropriate secondary antibodies conjugated with Texas Red were used for 2 h at 37°C (1:400; Texas Red Goat Anti-Rabbit IgG, **Cat. No. TI-1000;** or Texas Red Horse Anti-Mouse IgG, **Cat. No. TI-2000**; Vector Labs). In control experiments, cells were stained with secondary antibodies only. The nuclei were labeled with DAPI, and the fluorescence images were collected with an FV1000 confocal laser-scanning microscope (Olympus).

### Statistical analysis

All results are presented as mean ± SD. Statistical analysis of the data was done using Student's t-test for unpaired samples, with * p<0.05 and **p<0.005 considered a statistically significant result.

## Results

### Colon cancer HTC116 and HTB37 cell lines express mRNA for several pituitary and gonadal glycoproteins

**Fig 1:**
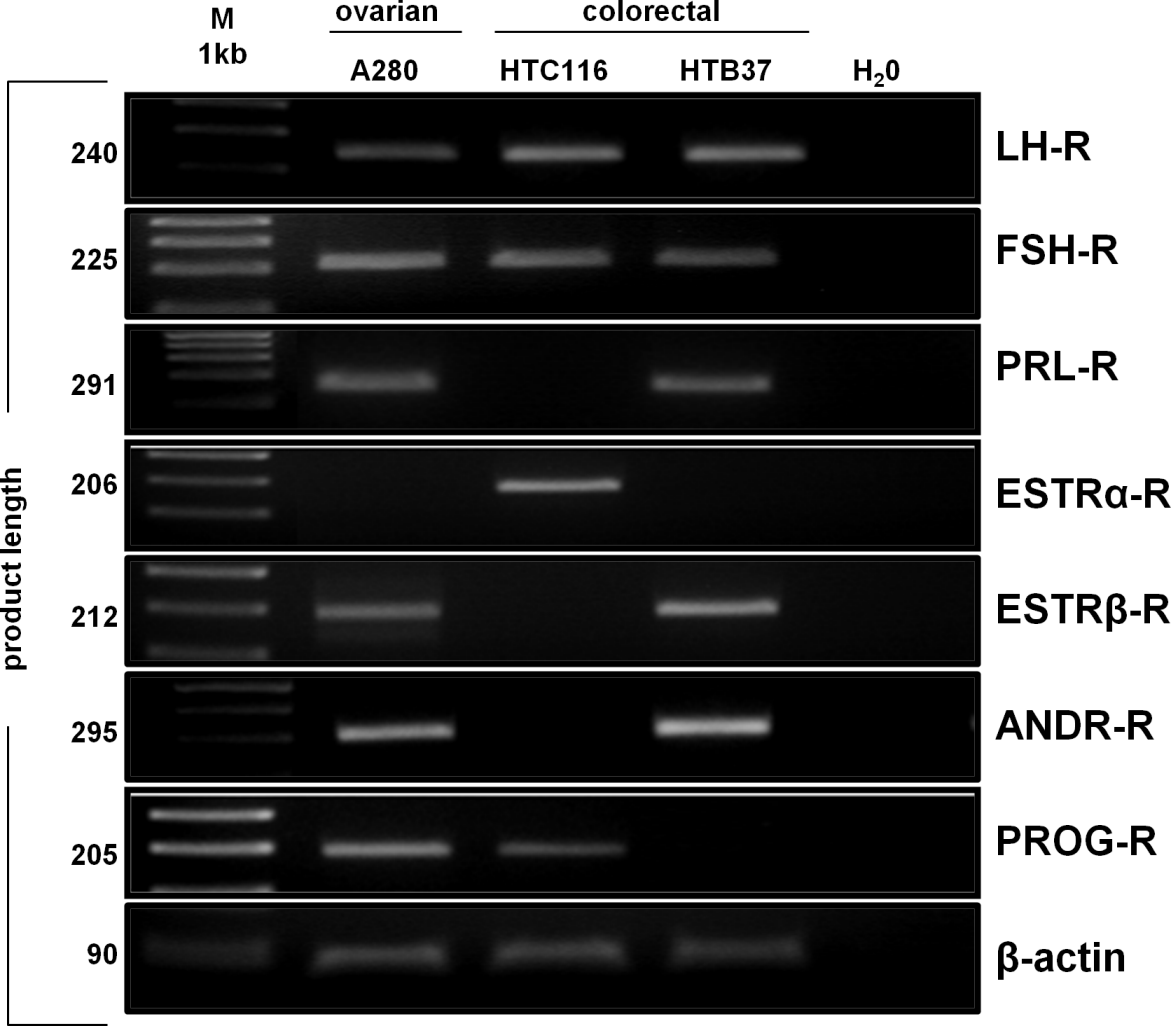
Pituitary gonadotropins and gonadal sex hormone receptor transcripts are expressed in the colon cancer cell lines HTC116 and HTB37. RT-PCR assays demonstrate the presence of PtGs and gonadal sex hormone RNA expression in selected colorectal cancer (CRC) cell lines. “H_2_O” indicates samples in which water was used instead of template. An ovarian cancer cell line (A280) served as positive control.

**Fig 2:**
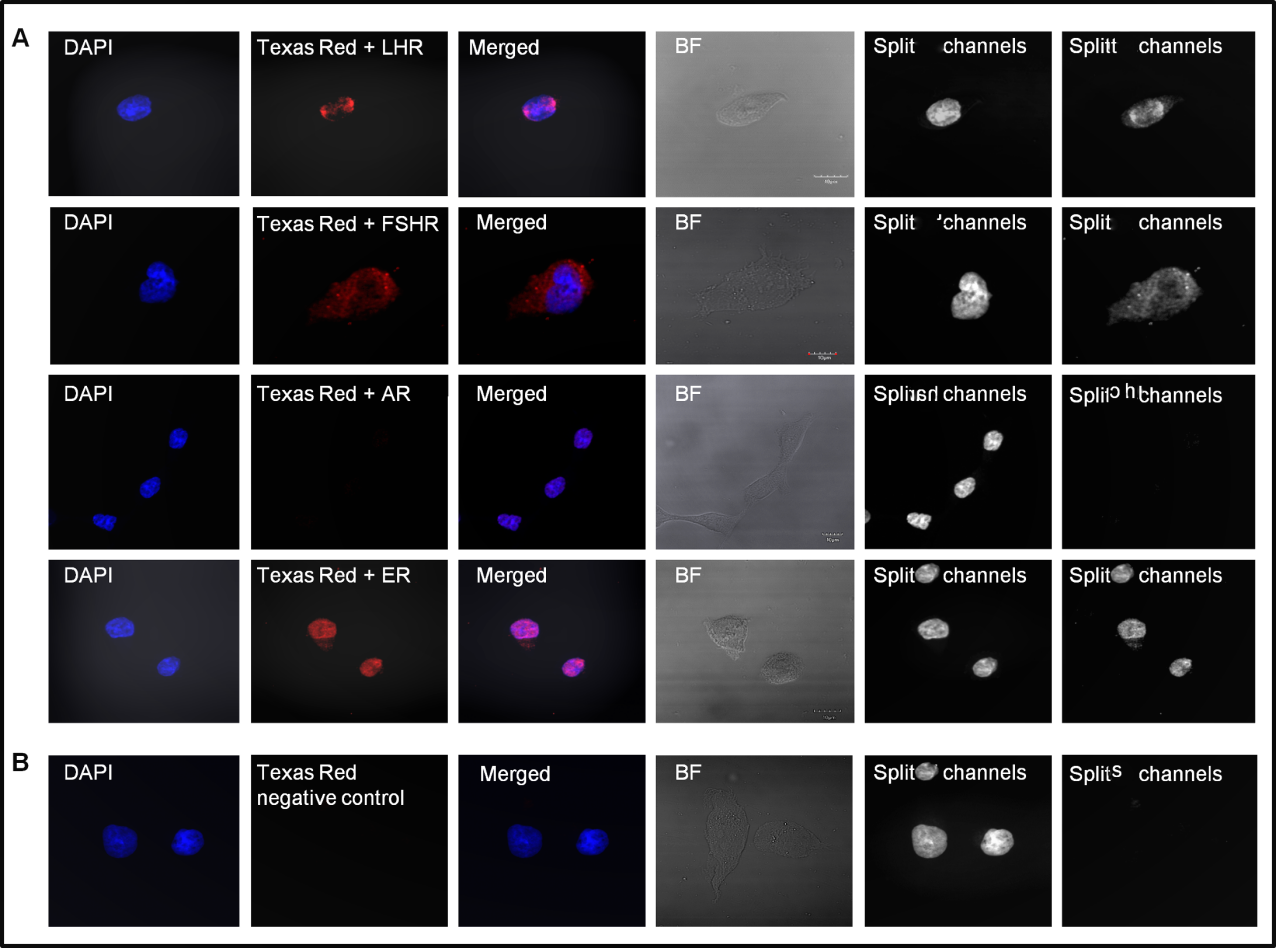
Human colon cancer cell line HTC116 expresses pituitary and gonadal glycoprotein receptors at the protein level. (A) Immunofluorescence staining of the transmembrane luteinizing hormone receptor (LH-R) and follicle-stimulating hormone receptor (FSH-R) and the nuclear androgen receptor (AR) and estrogen receptor (ER) in HTC116 cells. (B) Negative control. BF, bright field. Scale of merged picture, 10 μM.

First, to evaluate mRNA levels of PtGs and gonadal SexHs in human colon cancer cell lines, we performed RT-PCR assays. As shown in Fig 1, we detected mRNA for several SexH receptors by conventional RT-PCR. The HTC116 and HTB37 cell lines evaluated in the present study expressed follicle-stimulating hormone receptor (FSH-R) and luteinizing hormone receptor (LH-R). Furthermore, we were able to detect expression of the progesterone receptor (PROG-R) and estrogen receptor alpha (ESTR-α) in HTC116 cells. However, we were not able to detect the mRNA of PROG-R and ESTR-α in HTB37 cells. By contrast, the HTB37 cell line was positive for the presence of mRNA for the prolactin (PRL-R) and androgen (ANDR-R) receptors as well as for estrogen receptor beta (ESTR-β-R). As a positive control, human ovarian A2780 (OV) cancer cells, which express all of the SexH receptors except ESTR-α, were used. Besides the detection of transmembrane receptor transcripts, we were also able to detect expression at the protein level by immunofluorescence staining. Fig 2 shows the expression of LH, FSH, and the nuclear androgen receptor (AR) and estrogen receptor (ESTR) in the established CRC cell line employed in our study.

### Pituitary gonadotropins induce cell motility and adhesion in colon cancer cell lines

**Fig 3:**
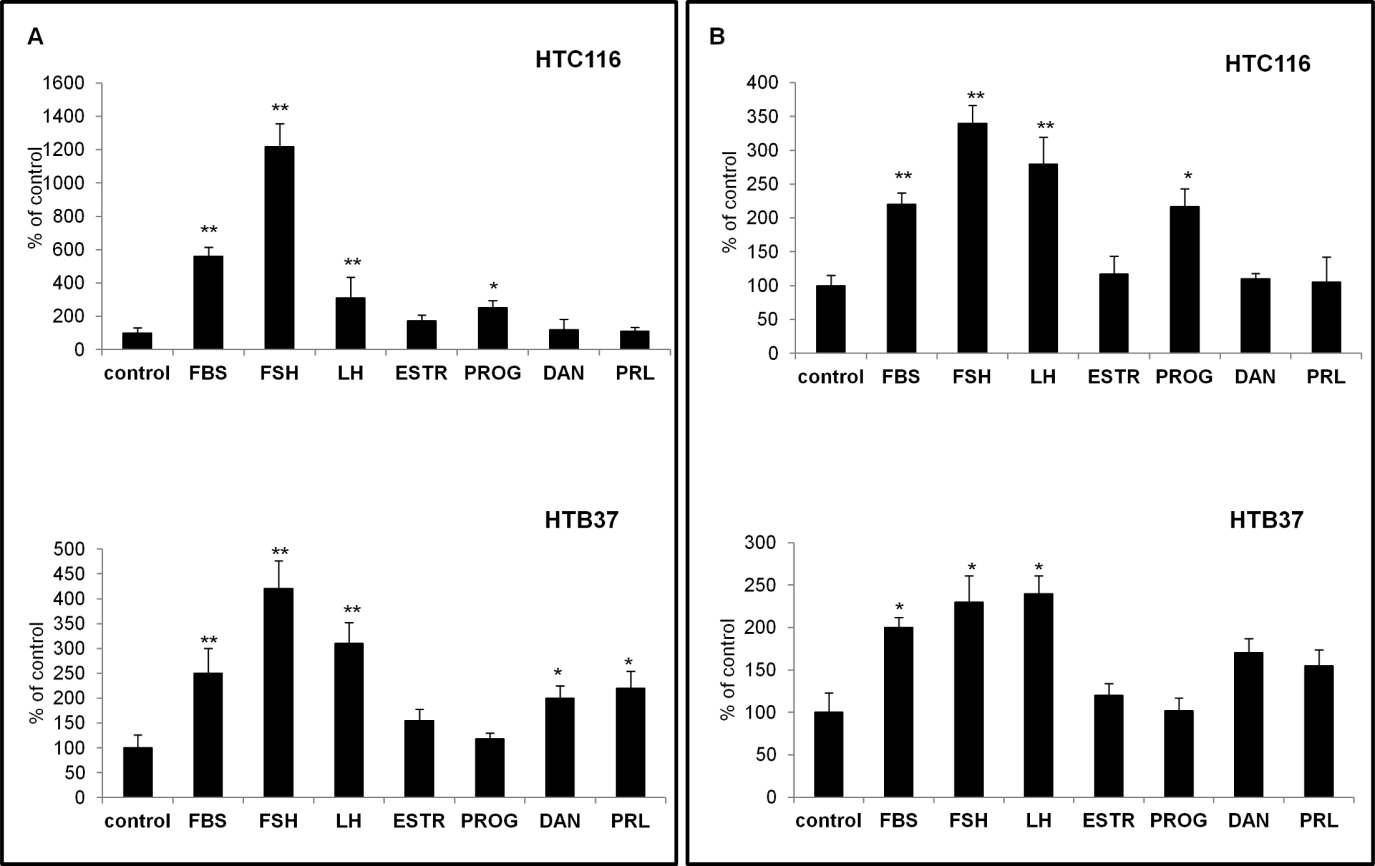
Effect of pituitary gonadotropins on migration and adhesion of CRC cell lines. (A) Migration of HTC116 and HTB37 cells in response to FBS (10%), follicle-stimulating hormone (FSH, 10 IU/ml), luteinizing hormone (LH, 10 IU/ml), estrogen receptor (ESTR, 100 nM), progesterone (PROG, 100 nM), danazol (DAN, 80 μg/ml), or prolactin (PRL, 0.5 μg/ml). *p≤0.05, **p≤0,005. (B) Adhesion of HTC116 and HTB37 cells after treatment with FSH (10 IU/ml), LH (10 IU/ml), ESTR (100 nM), PROG (100 nM), DAN (80 μg/ml), or PRL (0.5 μg/ml) to fibronectin-coated plates. The hormones were tested in serum-free medium. Bars indicate standard deviations; *p≤0.05, **p≤0.005.

Next, we tested whether PtGs are functional in colon cancer cell lines by performing chemotaxis and cell adhesion assays. In Transwell chemotaxis assays, we employed PtGs at the indicated high doses to test their chemotactic effects against selected cell lines. We found that, of all the sex hormones tested, the strongest chemotactic activity was observed for FSH (10 IU/ml) and/or LH (10 IU/ml), but cells in established CRC cell lines also responded to stimulation by other hormones (Fig 3A). Moreover, as in the chemotaxis assays, the HTC116 and HTB37 human CRC cell lines responded to SexH stimulation in fibronectin adhesion assays (Fig 3B).

### Pituitary gonadotropins may induce proliferation of colon cancer cells

**Fig 4:**
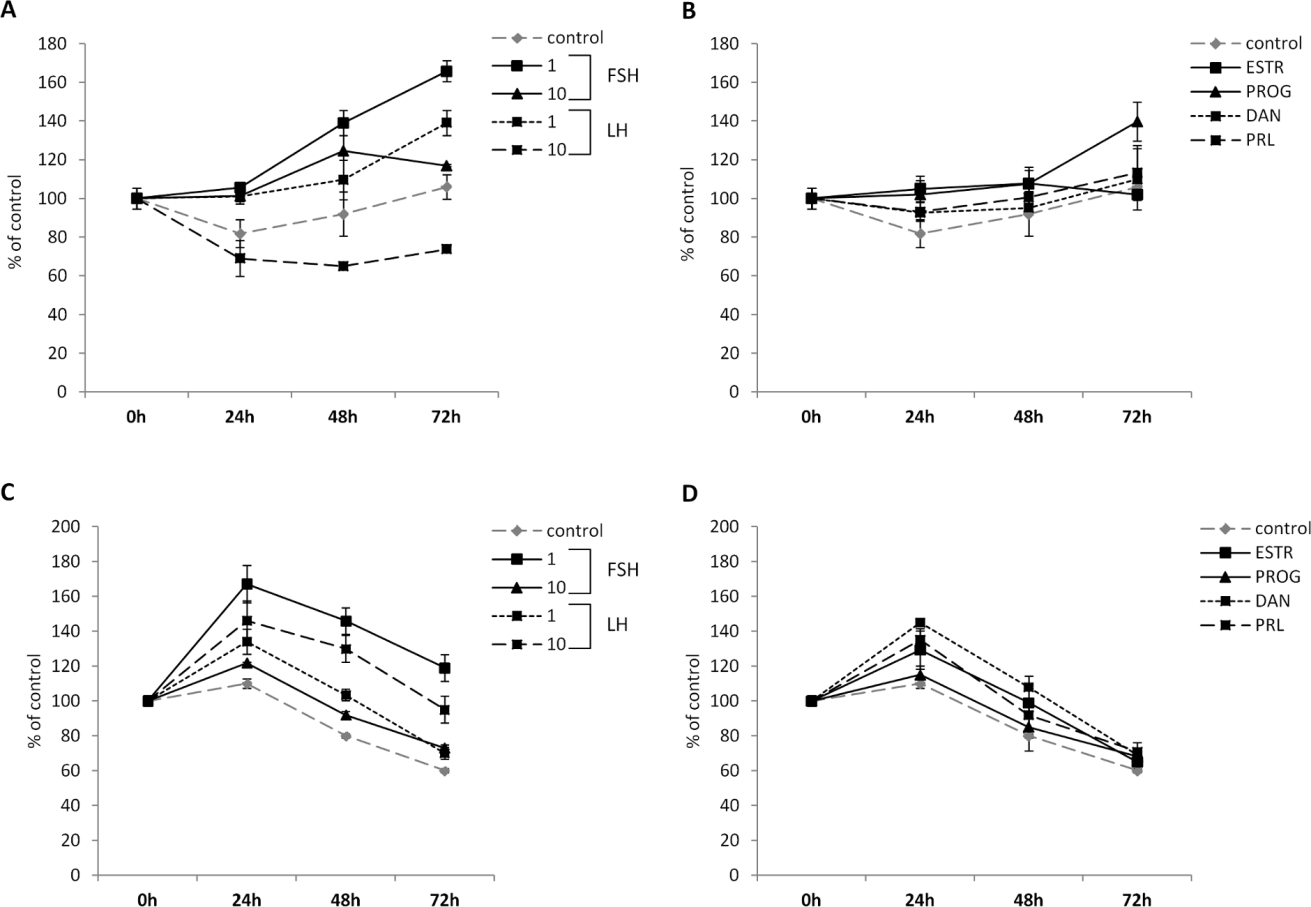
**Effect of pituitary and gonadal glycoproteins on the proliferation of HTC116 (A, B) and HTB 37 (C, D) cells.** Proliferation of HTC116 cells after treatment with (A) follicle-stimulating hormone (FSH, 1 and 10 IU/ml) or luteinizing hormone (LH, 1 and 10 IU/ml) or (B) estrogen receptor (ESTR, 100 nM), progesterone (PROG, 100 nM), danazol (DAN, 80 μg/ml), or prolactin (PRL, 0.5 μg/ml). Proliferation of HTB37 cells after treatment with (C) FSH (1 and 10 IU/ml) or LH (1 and 10 IU/ml) or (D) ESTR (100 nM), PROG (100 nM), DAN (80 μg/ml), or PRL (0.5 μg/ml).

**Fig 5:**
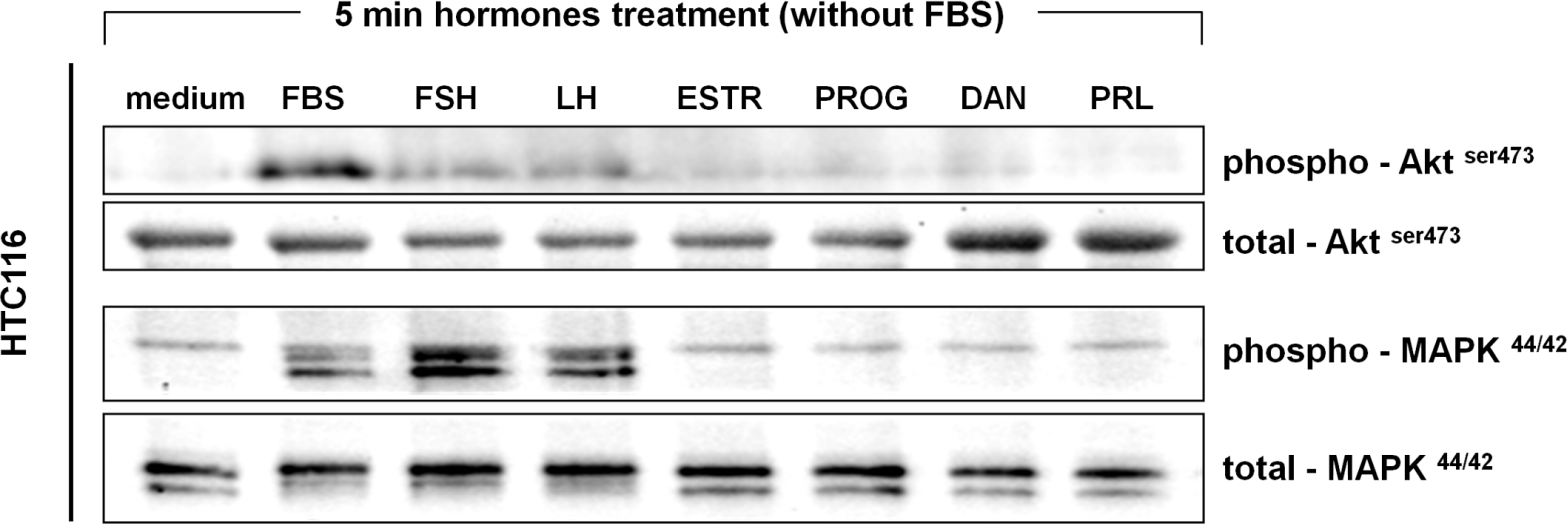
Phosphorylation of the intracellular kinase p42/44 mitogen-activated protein kinase (p42/44 MAPK) and AKT in HTC116 cells. The experiment was repeated twice with similar results, and representative data are shown. ll studied hormones were tested in serum-free medium (without FBS).

PtGs and gonadal SexHs have been reported to stimulate proliferation of both normal and malignant cells. To evaluate the effect of these hormones on colon cancer cell proliferation, we exposed HTC116 and HTB37 cells to all of the PtGs (Fig 4A and 4C) and gonadal SexHs (Fig 4B and 4D) evaluated in the present study. We observed that all of them stimulate proliferation of both CRC cell lines after a 24-h incubation. This stimulatory effect was further observed for up to 72 hours in the HTC116 but not in the HTB37 cell line. Furthermore, we found that the strongest proliferation activity of selected cells was observed for low doses of FSH (1 IU/ml) and/or LH (1 IU/ml). This effect seems to be direct, as the SexH receptors expressed by CRC cells responded to stimulation by phosporylation of MAPK^p42/44^ and AKT^ser473^ (Fig 5).

### Detection of mRNA for pituitary gonadotropins and gonadal sex hormone receptors in patient primary colon cancer samples

**Fig 6:**
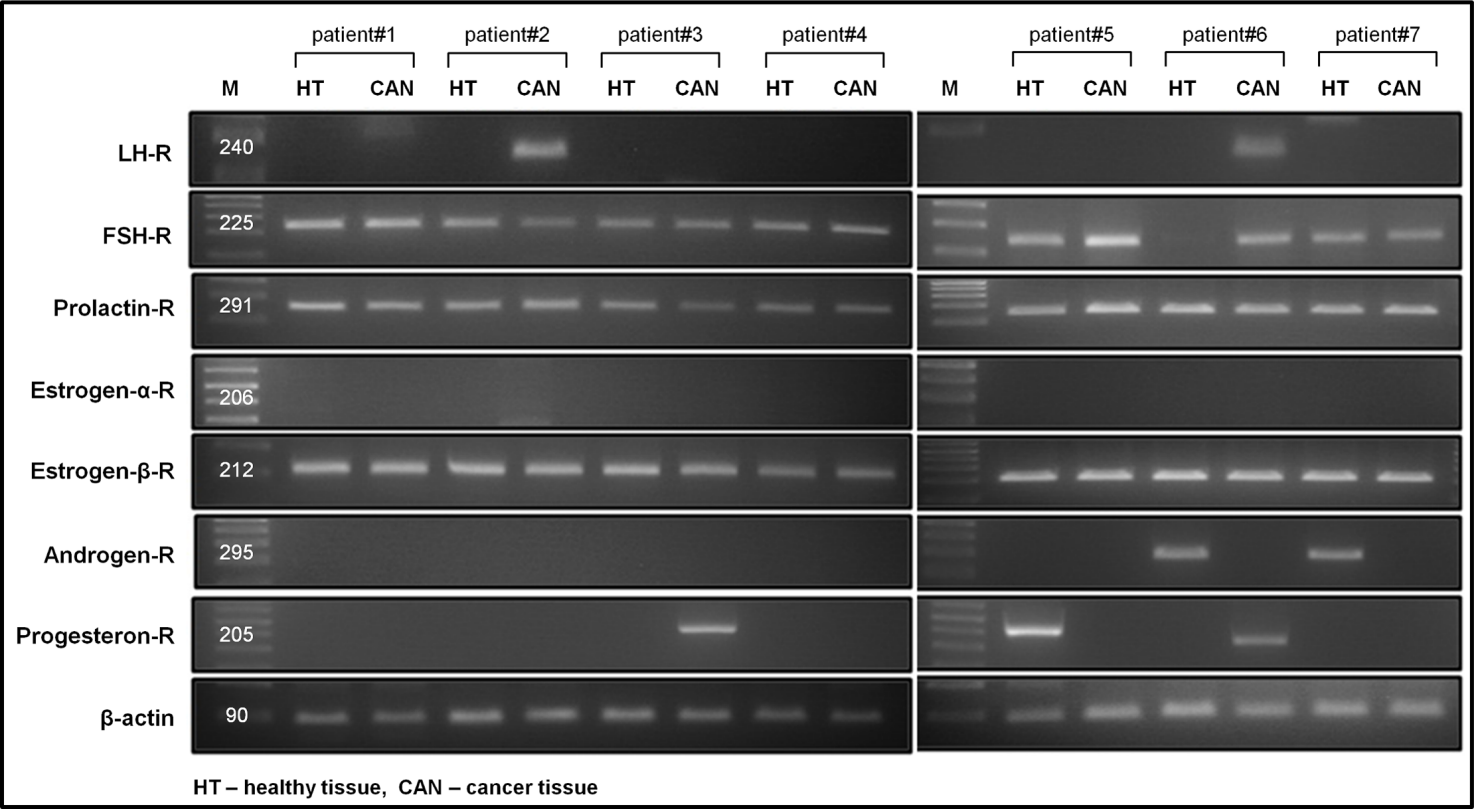
Pituitary gonadtropins and gonadal sex hormone receptors are expressed in colon cancer patient samples. RT-PCR assays demonstrate SexH RNA expression in healthy human colon tissue (HT) and tumor colon tissue (CAN) in seven cases (n = 7). The assay was performed three times with similar results. A representative gel image is shown.

Finally, we evaluated the expression of pituitary and gonadal SexH receptors in seven (n = 7) primary patient colon tumor samples. We obtained colon samples from both healthy and cancerous regions to compare the results. All patients were diagnosed with CRC adenocarcinoma in various stages of disease. Detailed patient characteristics and clinical histories are presented in Table 1. We detected FSH-R, PRL-R, and ESTR-β-R expression in almost all samples. As an example of an analyzed sample obtained from patient #6, FSH-R expression was absent in healthy colon tissue (Fig 6). Of note, as loss of FSH-R expression was found in healthy colonic biopsies in 1 of 7 individuals studied, it is difficult to draw conclusions about the biological significance of this phenomenon. Furthermore, we were able to observe the expression of LH-R only in colon cancer tissues of two patients (n = 2, patients 2 and 6). These patients (male and female) had the shortest survival time, since they were diagnosed with advanced disease with distant metastases. Expression of ANDR-R was present only in healthy colon tissue (n = 2, patients 6 and 7) and not in colon cancer samples. Moreover, we did not detect the presence of estrogen receptor alpha (ESTR-α-R) mRNA in any of the patient samples tested. PROG-R RNA was detectable in cancerous samples of patients #3 and #6 and in the healthy tissue of patient #5. These results correlated with our results obtained from established CRC cell lines (Figs 1 and 2).

**Table 1:** Demographic data for patients diagnosed with colorectal cancer.

CRC patients	1	2	3	4	5	6	7
Age	35	68	66	68	66	74	82
Gender	Male	Male	Male	Male	Male	Female	Female
BMI (kg/m^2^)	29	24	25	30	23	27	20
Histopatology*	G2	G2, mucocellulare	G2	G2	G2	G2	G1
Location (colon)	descending	cecum	rectum	sigmoid	descending	sigmoid	sigmoid
Staging (WHO)	pT3N0Mx	pT3N3M1	pT3N0Mx	pT3N1Mx	pT3N1Mx	PT3N3M1	pT1N0Mx
LVI	0	N/A	0	N/A	1	0	0
Co-morbidities	none	hypertension	none	none	hypertension	none	teratoma adultum (ovary)
Follow-up	ongoing	lost to death	ongoing	ongoing	ongoing	lost to death	ongoing
Survival since diagnosis	15 month	10 month	14 month	13 month	11 month	2 month	17 month

* Adenocarcinoma; BMI–body mass index, CRC–colorectal cancer, WHO–World Health Organisation; LVI–lymphovascular invasion; N/A–data not available

## Discussion

The seminal observation of our work is that cells from established human CRC cell lines, as well as tissues samples from patients suffering from CRC, express several PtGs and gonadal SexH receptors. Moreover, we provide for the first time evidence that FSH and LH stimulate proliferation, adhesion, and chemotaxis of human CRC cells.

Epidemiological data suggests that female SexHs play an important protective role in the etiology and progression of CRC [12–13, 28–30]. In support of such a role, exogenous administration of estrogen and progestin during the development of CRC in postmenopausal woman has been reported in several studies [31], and this phenomenon could be explained, as we hypothesize here, by a negative feedback of these hormones on PtG release, including of FSH [32]. By contrast, older, post-menopausal females have a worse overall survival prognosis than their male counterparts of a similar age [9,10]. This may correlate with a higher level of FSH observed in postmenopausal women in response to age-related decrease in gonadal function and impaired secretion of estrogens [11].

Overall, evidence has accumulated that PtGs and gonadal SexHs play a role in several female and male reproductive tumors, including breast, ovarian, and prostate cancer [33,34], but also in other non-gonadal malignancies, such as stomach and lung cancers [35–37]. For example, an increased risk of gonadal tumors, but not for stomach or CRC cancers, has been associated with the use of postmenopausal hormone replacement therapy (HRT). By contrast, modulation of the pituitary–gonad axis by means of estrogen administration was correlated with a decreased risk of certain gastrointestinal tumors [37]. In our previous reports we demonstrated the presence of functional SexH receptors in human lung cancer [16], rhabdomyosarcoma [21], and leukemia cells [22].

In contrast to other malignancies, data on pituitary and gonadal hormone receptor expression in CRCs are scarce. Huang *et al*. demonstrated the presence of luteinizing hormone-releasing hormone (LH-RH) and its receptor immunoreactivity in the rat small and large intestinal epithelium [38]. Szepeshazi *et al*. [39] documented the presence of high-affinity binding sites for LH-RH in several established CRC cell lines and showed the potential of their functional inhibiton by LH-RH analogs.

In our study we report the novel observation that FSH, LH, but also PRL and DAN, are potent mitogens for human CRC cells. This observation is important and has clinical implications. Since the incidence of CRC worldwide is reported to be rising, the increase in levels of pituitary SexHs (FSH and LH) in patients due to age-related gonadal dysfunction requires more attention.

We demonstrate for the first time that PtGs stimulate migration, adhesion, and proliferation of established human CRC cell lines. In our experiments we observed the expression of PtGs and gonadal SexH receptors in established human CRC cell lines, not only at the mRNA level but also at the protein level. Moreover, PtGs mediate this effect by their specific receptors, which respond by the phosphorylation of intracellular pathways involved in cell proliferation and migration. Finally we detected SexH receptor expression in CRC patient samples and demonstrated SexH mRNA expression in healthy human colon tissue adjacent to the tumor (HT) and tumor colonic tissue (CAN).

Recently, tremendous effort has been made to better understand the mechanisms of CRC development and its metastasis. Current concepts revolve around stem cells [40, 41], the role of innate immunity [42,43], and alterations in the microbiome [44]. Our observation that SexH receptors are expressed not only in CRC cells but, what is more important, also in CRC colonic patient samples sheds new light on this phenomenon. Thus, pituitary and gonadal hormones may be involved in the egress of cancerous cells into blood or lymphatic vessels and their migration to distinct locations. In fact, we demonstrated the effects of SexHs on the migration, adhesion, and proliferation of CRC cells.

Moreover since the follicle-stimulating hormone receptor FSH-R has been shown to be expressed by the vascular endothelium in several human cancers [45] as well as in tumor metastases [46], including colon cancer [47], the detection of SexH receptors by RT-PCR may reflect the expression of SexH receptors on the surface of tumor-associated cells of the vasculature. In fact, Alam *et al*. [48] showed that binding of FSH to the FSH receptor in ovarian granulosa cells induced an increase in hypoxia-inducible factor 1α protein levels and led to up-regulation of vascular endothelial growth factor (VEGF). This and other observations provide evidence that FSH receptor expression can induce VEGF and VEGF receptor 2 (VEGFR-2) signaling in endothelial cells, promoting tumorigenic angiogenesis [49,50]. However, this effect could be local and limited to epithelium, as we did not observe differences in plasma VEGF concentrations between CRC patients and healthy controls in our previous reports [40]. In contrast to our previous study, we observed higher levels of hepatocyte growth factor (HGF) in the plasma of patients diagnosed with CRC [40]. Of interest, the reported action of FSH and LH on ovarian surface epithelium (OSE) growth could in part be mediated indirectly through an elevation in the expression of autocrine growth factors, such as HGF [51].

On the other hand, according to the hypothesis proposed by Virchow, Conheim, and Boll, some cancers originate from dormant embryonic or germ cells residing in adult tissues [52–54]. These cells could be activated locally or could be attracted from a population of circulating SCs [55]. However, this tempting 150-year-old hypothesis requires further experimental support to identify the cells that are the origin of these tumors. Potential candidate cells located in adult tissues could be very small embryonic-like stem cells (VSELs) [56,57], which have been shown to express several SexH receptors [27,32] Thus, it is important to explore the possibility that mutated VSELs can give rise to CRC stem cells. In support of a contribution by circulating SCs, Houghton *et al*. [58] observed that bone marrow-derived stem cells (VSELs?) repopulated the gastric epithelium in response to chronic inflammation and in some cases contributed to malignant transformation and the development of stomach cancer. Nevertheless, both of these hypotheses need to be validated for CRC by further experimentation.

We are aware, that the current study has limitations. First, we used only two CRC cell lines, and the colonic samples were obtained from only 7 patients. Second, it has yet to be confirmed whether the biological effects of FSH and LH are truly mediated by the FSH and LH receptors. Further studies with siRNA/shRNA knockdown or CRISPR-Cas9-mediated knockout of receptors in cell lines should be implemented. We are currently implementing functional studies to fully explore this issue and exclude possible non-canonical actions of gonadotropins in healthy and cancerous colonic tissues.

In conclusion, our results provide novel evidence supporting a link between PtGs and CRC. Since FSH levels increase with age due to loss of gonadal function, this evidence may explain the age-related increase in this and other malignancies. Our hypothesis is somewhat supported by epidemiological observations of lower CRC risk in women on long-term estrogen replacement therapy, which lowers secretion of FSH from the pituitary.

In this light, further studies that could lead to development of new therapeutic protocols, including compounds capable of balancing the effects of PtGs and gonadal SexH axes, are necessary. Similarly, further work is needed to better understand the regulation of pituitary and gonadal hormone receptors on the surface of cancer cells. While PtGs are well known to upregulate expression of estrogen receptors in several types of cells [59], the potential hormonal up-regulators of PtGs have not yet been identified.
