# Expression of Concern: A novel potential role of pituitary gonadotropins in the pathogenesis of human colorectal cancer

**DOI:** 10.1371/journal.pone.0259176

**Published:** 2021-10-21

**Authors:** The PLOS ONE Editors

Following publication of this article [1], concerns were raised about some of the gel panels in Fig 1.

Specifically,

- Lanes 3 and 4 of the LH-R panel appear similar
- Lanes 2–4 of the ESTRα-R panel appear similar to lanes 3–5 of the ESTRβ-R panel
- In the PRL-R panel and the ESTRα-R panel there is an appearance of vertical discontinuity between lane 1 (marker lane) and lane 2

The corresponding authors noted that the original image files underlying Fig 1 are no longer available. The corresponding authors stand by the published data and commented that the observed similarities in band shapes resulted from the indicated samples having been run on the same gel. They acknowledged that lanes were re-ordered and spliced together from the same gels in preparation of some of the figure panels, in particular to place the marker consistently in lane 1. Additionally, the ESTRα-R and ESTRβ-R samples were run on a single gel; one photograph was taken of the marker and ESTRβ-R lanes, then a separate photograph was taken of the ESTRα-R lanes, which were spliced together to the marker lane for preparation of the figure panel.

The corresponding authors provided replicate gel images for some of the RT-PCR assays; however, editorial evaluation determined that these replicate gels were not adequate to fully support the original results. The corresponding authors have also repeated the experiment to provide a revised Fig 1 accompanied by all underlying gel image files (S1 File). The data for the repeat experiments appear to support the published results.

There is an error in the primer sequences reported in the Methods section for hLH-R. The correct primer sequences are: F, 5’-CCGGTCTCACTCGACTATCAC and R, 5’-TGAGGAGGTTGTCAAAGGCA.

In the published Fig 1, there is an error the name of the positive control ovarian cancer cell line in the figure labels and figure caption. The correct positive control ovarian cancer cell line label is A2780.

The Competing Interests statement is amended to include patent application information to meet the requirements of the PLOS Competing Interests policy, as follows:

At the time of article publication, MZR is named as one of several inventors on a patent application owned by University of Louisville entitled ““Very small embryonic-like stem cells and methods of isolating and using the same”. MZR considers that the patent is related to a different topic than the *PLOS ONE* article. The other authors have declared that they have no competing interests. This does not alter our adherence to *PLOS ONE* policies on sharing data and materials.

The *PLOS ONE* Editors issue this Expression of Concern to make readers aware of concerns about the figure preparation practices and the reliability of the image data for Fig 1, which cannot be fully resolved because of the unavailability of the original image files underlying Fig 1.

All underlying data for the rest of the article are available upon request to the corresponding authors.

## Supporting information

S1 File Repeat experiment gels and revised Fig 1.zip. (ZIP)

## References

[1] Marlicz W, Poniewierska-Baran A, Rzeszotek S, Bartoszewski R, Skonieczna-Żydecka K, Starzyńska T, et al. (2018) A novel potential role of pituitary gonadotropins in the pathogenesis of human colorectal cancer. PLoS ONE 13(3): e0189337. 10.1371/journal.pone.0189337 29494614 PMC5832186

