# Supplementary figures and images for: Expression of Concern: A novel potential role of pituitary gonadotropins in the pathogenesis of human colorectal cancer
Source: PLoS One. 2021 Oct 21;16(10):e0259176. doi: 10.1371/journal.pone.0259176 (PMC8530320; doi:10.1371/journal.pone.0259176)

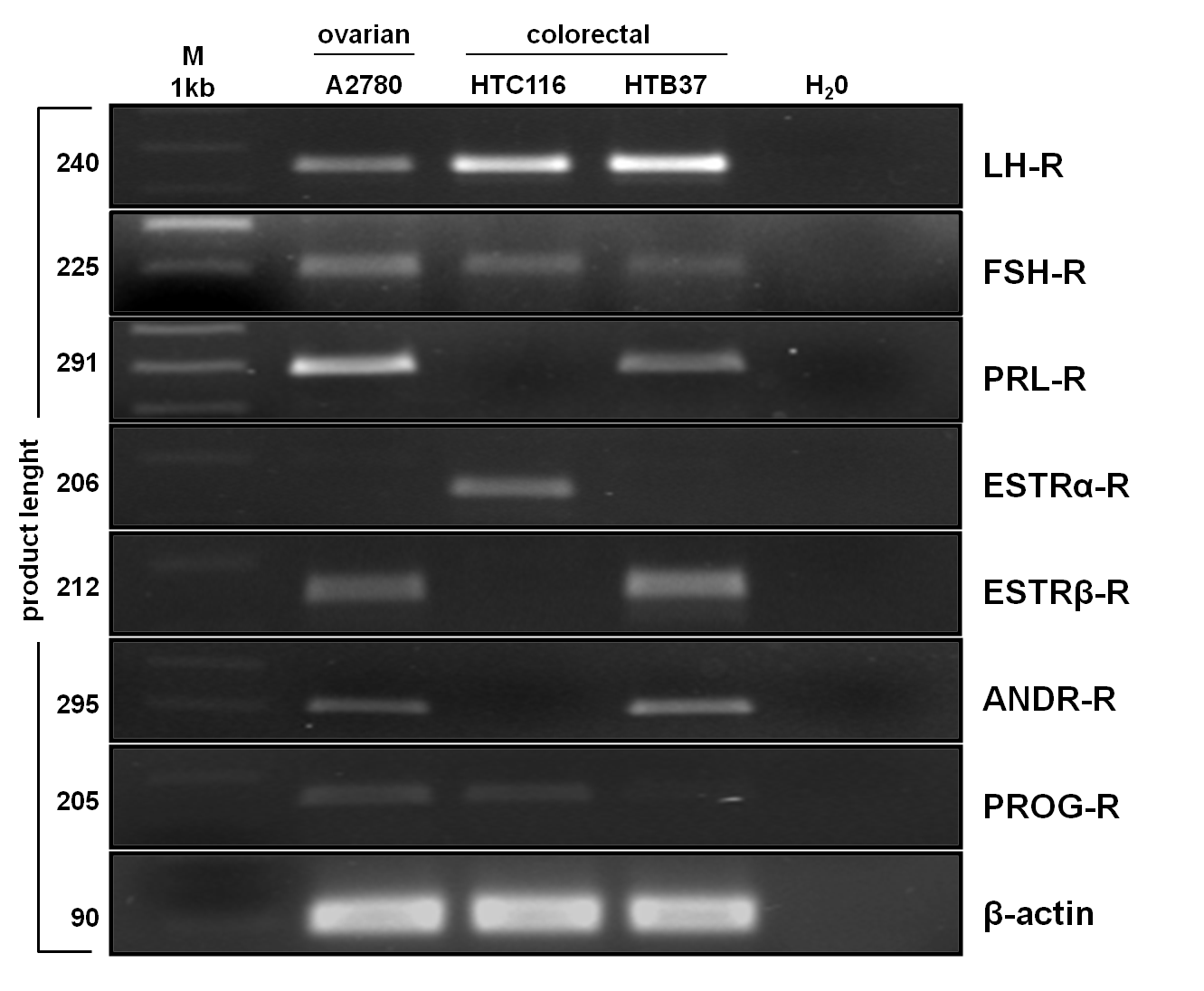

Supplement: S1 File — (ZIP) [file pone.0259176.s001.zip › Fig1_multi_panel_receptors.tif]
